# Downregulation of HLA-ABC expression through promoter hypermethylation and downmodulation of MIC-A/B surface expression in LMP2A-positive epithelial carcinoma cell lines

**DOI:** 10.1038/s41598-020-62081-0

**Published:** 2020-03-25

**Authors:** Shweta Singh, Subrata Banerjee

**Affiliations:** Biophysics and Structural Genomics Division, Saha Institute of Nuclear Physics, Homi Bhabha National Institute, Kolkata, India

**Keywords:** Tumour immunology, Immunoediting

## Abstract

Epstein Barr Virus (EBV) is a human herpesvirus, and has been reported to be associated with nasopharyngeal carcinoma, gastric carcinoma, Burkitt’s lymphoma and Hodgkin’s lymphoma. In most of the associated tumors, the virus remains in a latently infected state. During latency, EBV expresses Latent Membrane Protein 2A (LMP2A) along with few other genes. We previously showed that LMP2A causes downregulation of HLA-ABC surface expression in EBV associated gastric carcinomas. However, the mechanism that leads to this downregulation remain unclear. We therefore analyzed methylation-mediated regulation of HLA-ABC expression by LMP2A. Interestingly, according to the ‘missing self’ hypothesis, when there is a decrease in HLA-ABC surface expression, expression of NKG2D ligands’ must be upregulated to facilitate killing by Natural Killer (NK) cells. Analysis of NKG2D ligands’ expression, revealed downregulation of MIC-A/B surface expression in response to LMP2A. Furthermore, the role of Unfolded Protein Response (UPR) in the regulation of MIC-A/B surface expression in cells expressing LMP2A was also investigated. Protein Disulfide Isomerase (PDI) mediated inhibition of MIC-A/B surface expression was observed in LMP2A expressing cells. Our current findings provide new insights in LMP2A arbitrated dysregulation of host immune response in epithelial cell carcinomas.

## Introduction

Host immune response plays pivotal role in development and progression of cancer. CTLs and NK cells play important role in recognition and elimination of virus-infected cells. Downmodulation of the HLA class I antigen processing pathway^1,2^ along with proteasome subunits act as strategies utilised by the viruses to overcome host immune response. Transporter associated with antigen presentation, β-microglobulin and HLA class-I heavy chains are also reported to be targeted during viral infection^3^. Alternatively, tumour immune evasion mechanism involves internalization and shedding of NKG2Dligands’, MHC class I chain-related proteins A and B (MIC-A and MIC-B) and UL16-binding proteins (ULBPs), ensuing inhibition of NK cell-mediated cytotoxicity^4,5^.

EBV, γ-human herpes virus, is known to be associated with various malignancies such as Burkitt’s Lymphoma, Hodgkin’s Lymphoma, Nasopharyngeal Carcinoma, Gastric Carcinoma, and Breast Cancer^6–9^. EBV-associated gastric carcinoma (EBVaGC), an epithelial cell origin carcinoma has recently gained importance^10^. EBV manifests life-long latent infection in most of the EBV-associated malignant neoplasm. EBV establishes latent infection in most of the tumors where it expresses Latent Membrane Protein 2A (LMP2A) along with other EBV-encoded genes^6^. The viral oncoprotein, LMP2A plays important role in the maintenance of latency and is recognized to be associated with transformation, anchorage, motility and differentiation in epithelial cells^11^. We previously reported LMP2A mediated increased cellular migration through alteration of mitochondrial dynamics^12^. LMP2A is an integral transmembrane protein, consisting of a long tyrosine rich 119 amino acid N-terminal cytoplasmic tail, along with 12 hydrophobic membrane-spanning domains and a short cytoplasmic tail of 27 amino acid in the C-terminus. Immunoreceptor tyrosine-based activation motif (ITAM) in the N-terminus consist of eight tyrosine residues along with proline and tyrosine rich motif (PY) and tyrosine, two-glutamic acid, alanine motif (YEEA)^13,14^. LMP2A is reported to constituitively activate PI3-kinase (PI3-k) and Akt signaling cascade^15^. Earlier studies show defective HLA Class I mediated antigen processing and presentation during EBV infection in Burkitt’s lymphoma along with nasal NK/T-cell lymphoma^16,17^.

Human leukocyte antigen (HLA) also referred to as Major Histocompatibility Complex (MHC) is a cell surface glycoprotein. HLA is reported to present intracellular peptides derived from viral and tumour antigens to the counteracting T-cell receptors, thus resulting in recognition of virus-infected tumour cells by CTLs. We previously reported decreased HLA-ABC surface expression through EBV latent protein, LMP2A in EBVaGC^18,19^. However, mechanisms responsible for HLA-ABC gene downregulation aside from its decreased surface-level expression in EBVaGC are yet to be fully investigated. Molecular research has provided information for regulation of gene expression based on epigenetic alterations. Epigenetic alteration includes DNA methylation-mediated regulation of gene expression which is executed by DNA methyltransferases (DNMTs)^20^. Ubiquitin-like with PHD and Ring Finger Domain 1 (UHRF1) is recently been identified to assist DNMT1 in hoisting methylation of a gene^21^. However, promoter methylation of the HLA-A, HLA-B and HLA-C gene in EBV associated epithelial cell carcinomas was never been studied previously. Therefore, in the present study, we analyzed the methylation status of HLA-A, HLA-B and HLA-C gene promoter region in LMP2A expressing epithelial cell carcinomas. To further validate the role of methylation in downregulated expression of HLA-ABC, demethylation study was performed using 5′-azacytidine in LMP2A expressing epithelial cell carcinomas. In addition to CTLs, Natural Killer (NK) cells play crucial role in providing early immune defense during viral infection according to the ‘missing self’ hypothesis’^22^. NK cell-mediated killing of virus-infected cells requires the expression of the activating receptor, NKG2D (natural killer group2, member D) on NK cells, NKT cells, and some CTLs. Eight tumour-associated ligands’ are identified for human NKG2D activating receptors which include MIC-A and MIC-B, along with six retinoic acid early transcript-1 proteins ULBPs (ULBP1-6). During NKG2D-mediated tumour recognition, down-regulation of NKG2D ligands’ expression suggests an important mechanism by which tumour cells escape recognition by immune cells^23,24^. We investigated the expression of NKG2D ligands’, primarily MIC-A and MIC-B. MIC-A/B surface expression in LMP2A expressing epithelial cell carcinomas was targeted, resulting in decreased susceptibility of the infected cell to NK cell-mediated cytotoxicity. Surface expression of NKG2D ligands’ is modulated by proteolytic cleavage in tumour cells resulting in elevated serum levels of soluble NKG2D ligands’^25,26^. To avoid NK cell activation, EBV has recently been shown to inhibit MIC-B translation through miR-BART2-5p^27^. Expression of NKG2D ligands’ is documented to be governed by cellular stresses^25^.

Accumulation of unfolded proteins in the ER results in induction of Unfolded Protein Response (UPR). The proteins which play an important role in maintaining UPR include Inositol-requiring transmembrane kinase/endoribonuclease 1α (iRE1-ɑ), Binding Immunoglobulin protein (BIP), C/EBP homologous protein (CHOP), Protein Disulfide Isomerase (PDI) along with other proteins^28,29^. UPR proteins frequently detected in EBV-infected Nasopharyngeal Carcinoma (NPC) resulted in upregulation of EBV oncoprotein, Latent Membrane Protein1 (LMP1)^30^. Disulfide isomerases are recently reported to induce shedding of tumour-associated NKG2D ligands’ thereby promoting immune evasion^31^. PDI, an UPR protein has dithiol-disulfide oxidoreductase activity through which it catalyzes the formation and isomerization of disulfide bonds^32,33^. PDI shuttles between cell cytoplasm and outer-membrane in certain tumors^34^. Membrane-bound PDI facilitates the reduction of disulfide bonds in cell surface proteins^35^. We investigated that loss of MIC-A/B surface expression was due to PDI. This comprehensive analysis of cell surface molecules in EBV associated epithelial cell carcinomas, provides insights into molecular mechanisms by which LMP2A regulates immune evasion.

## Results

### Decreased expression of HLA-ABC by EBV LMP2A

**Figure 1:**
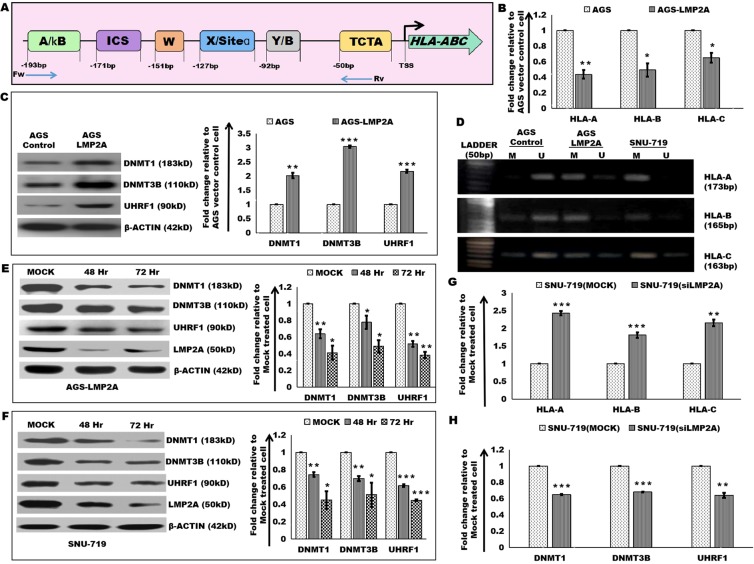
Decreased expression of HLA-ABC by EBV-LMP2A. (**A**) Schematic representation of the HLA-ABC promoter region showing the enhancer sites, A/kB enhancer (−193 to −181), ICS (−171 to −159), W box (−151 to −145), X/Siteα box (−127 to −110), enhancer B (−92 to −87) and TATA box (TCTA). The blue arrows indicate the Forward (Fw) and Reverse (Rv) primers used to amplify the methylated region within the promoter site. (**B**) qRT-PCR of HLA-A, HLA-B, and HLA-C in AGS-LMP2A relative to AGS vector control cell. (**C**) Immunoblotting experiments to determine DNMT1, DNMT3B, and UHRF1 levels in AGS Control and AGS-LMP2A. The bar graph depicts the densitometry quantification of relative expression levels of DNMT1, DNMT3B, and UHRF1. (**D**) Methylation Specific PCR analysis of the human leukocyte antigen (HLA) class Ia gene promoter in AGS, AGS-LMP2A, and SNU-719 cells. Genomic DNA was treated with sodium bisulfite and amplified with specific primers for methylated (M) and unmethylated (U) alleles. (**E**) Immunoblotting experiments to determine DNMT1, DNMT3B and UHRF1 levels upon siRNA mediated knockdown of LMP2A expression in AGS-LMP2A cells at 48 and 72 hr compared to Mock treated cells. (**F**) Protein level measurement of DNMT1, DNMT3B and UHRF1 in SNU-719 cells upon siRNA mediated knockdown of LMP2A expression at 48 and 72 hr compared to Mock treated cells. (**G**) qRT-PCR of HLA-A, HLA-B and HLA-C in SNU-719 cell (siRNA-LMP2A) compared to Mock treated cell. (**H**) qRT-PCR of DNMT1, DNMT3B and UHRF1 in SNU-719 cell (siRNA-LMP2A) compared to Mock treated cell. Results are represented as mean ± s.e.m. of triplicate experiments. Data represent an average of n = 3 independent experiments. *P < 0.05, **P ≤ 0.01, ***P ≤ 0.001.

EBV has been shown to stably alter gene expression through aberrant DNA methylation in EBV infected epithelial cells^36^. EBV latent protein, LMP2A cDNA was cloned within the EcoR1 site of the pcDNA 3.1 vector and stably transfected into EBV-negative gastric cancer cell, AGS. Clones which showed expression pattern of LMP2A similar to that of EBV-positive Korean gastric cancer cell, SNU-719 were selected for the study^12,19^. Protein level measurement of LMP2A expression was estimated in AGS and AGS-LMP2A cells (Supplementary Fig. 1A). A schematic description of HLA-ABC promoter region is provided with enhancers sites (Fig. 1A)^37^. Transcription Factors (TFs) including Nuclear Factor kappa-light-chain-enhancer of activated B cells (NFkB), Class II major histocompatibility complex Transactivator (CIITA), and Regulatory Factor X (RFX5) are reported to bind the promoter region at the enhancer sites. We previously showed decreased protein expression of the above-mentioned transcription factors in LMP2A expressing gastric cancer cells^19^. A decrease in transcript-level expression of HLA-ABC in gastric cancer cell, AGS expressing LMP2A gene was evaluated through quantitative Real-Time PCR (qRT-PCR) (Fig. 1B). Since epigenetics play important role in regulation of gene expression, we investigated the role of epigenetic regulators in the regulation of HLA-ABC gene expression. First we identified the epigenetic regulators with increased expressions in, AGS expressing LMP2A (AGS-LMP2A) through quantitation of mRNA level compared to vector control cells (AGS) using qRT-PCR. An up-regulated expression of DNMT1, DNMT3B, and UHRF1 at transcript level was observed (Supplementary Fig. 1B), further validated by immunblotting experiments (Fig. 1C). Next, we explored methylation status associated with repressive levels of HLA-ABC gene. MSP^38^ was performed at the promoter region of HLA-A, HLA-B and HLA-C. Promoter hypermethylation was detected in EBV-positive gastric cancer cell, SNU-719, and AGS-LMP2A cells (Fig. 1D). The involvement of epigenetic regulators was further validated through immunoblotting experiments in AGS-LMP2A and SNU-719 cells upon 48 hr and 72 hr post-knockdown of LMP2A through siRNA. Decreased protein level expression of DNMT1, DNMT3B and UHRF1 in a time-dependent manner was detected in response to siRNA mediated targeting of LMP2A (Fig. 1E,F). Transcript level expression of HLA-ABC (Fig. 1G) and epigenetic regulators (Fig. 1H) was further validated in SNU-719 cells expressing LMP2A gene and SNU-719 cells treated with siRNA for LMP2A. Downregulated expression of HLA-ABC and epigenetic regulators was observed upon targeting of LMP2A.

### LMP2A Mutants were screened for HLA-ABC expression

**Figure 2:**
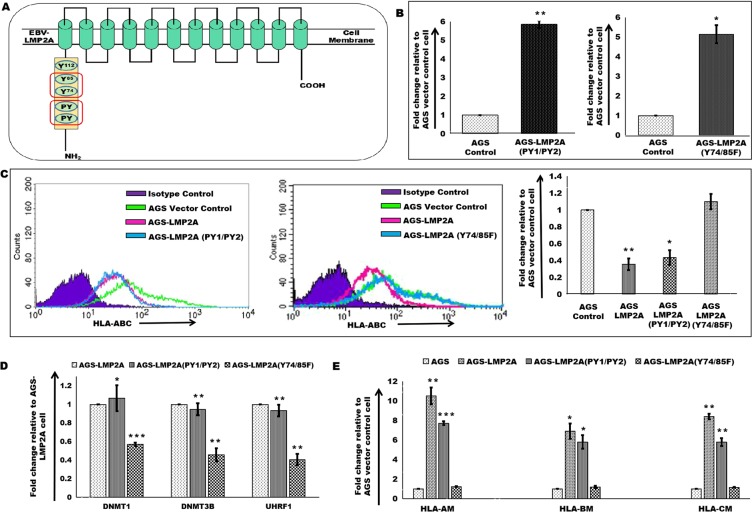
LMP2A Mutants were screened for HLA-ABC expression. (**A**) Schematic diagram of EBV latent membrane protein, LMP2A. The mutated regions in the ITAM are marked with the red box. (**B**) qRT-PCR of LMP2A(PY1/PY2) and LMP2A(Y74/85 F) in AGS cells stably expressing the mutated genes, AGS-LMP2A(PY1/PY2) and AGS-LMP2A(Y74/85F), respectively, relative to AGS vector control cell. (**C**) HLA-ABC surface expression analysis through FACS, upon introduction of mutant LMP2A (PY1/PY2) and LMP2A(Y74/5F) into AGS cells relative to a vector control cell. Quantitation of results was performed by measuring median values using Cell Quest Pro software. (**D**) Immunoblotting experiments to determine DNMT1, DNMT3B and UHRF1 levels in AGS LMP2A (PY1/PY2) and AGSLMP2A (Y74/85F) relative to AGS-LMP2A cells. (**E**) qRT-PCR of bisulfite modified genomic DNA template using MSP primers for amplification of methylated regions of HLA-A, HLA-B and HLA-C promoter in AGS-LMP2A, AGS-LMP2A(PY1/PY2) and AGS-LMP2A(Y74/85F) compared to AGS vector control cell. Results are represented as mean ± s.e.m. of triplicate experiments. Data represent an average of n = 3 independent experiments. *P < 0.05, **P ≤ 0.01, ***P ≤ 0.001.

LMP2A acts as an important viral protein for the maintenance of latent infection. LMP2A consists of an ITAM region similar to B-Cell Receptor which includes eight tyrosine^14^. Two such mutants 1) PY1/PY2 and 2) Y74/85F were designed using ‘Site-directed mutagenesis’ in the ITAM region of LMP2A (Fig. 2A). Mutated versions of LMP2A, pcDNA3.1/LMP2A-PY1/PY2 [LMP2A(PY1/PY2)] and pcDNA3.1/LMP2A-Y74/85F [LMP2A(Y74/85F)] were stably introduced into AGS cells and were selected using G418. The transcript level expression of mutant LMP2A genes was also quantitated in AGS-LMP2A(PY1/PY2) and AGS-LMP2A(Y74/85F) compared to AGS cells expressing pcDNA3.1 (AGS) (Fig. 2B). The expression of mutant versions of LMP2A in AGS cell was validated at the protein level through immunoblotting experiments (Supplementary Fig. 1C). Clones showing similar expression pattern as AGS-LMP2A were used in the study. HLA-ABC surface expression was also quantitated in AGS-LMP2A(PY1/PY2) and AGS-LMP2A(Y74/85F) compared to AGS cells (Fig. 2C). HLA-ABC surface expression was further validated in HEK293 cells transiently expressing LMP2A and mutated versions of LMP2A (Supplementary Fig. 2C). Decreased surface expression of HLA-ABC was verified in AGS and HEK293 expressing LMP2A and mutated LMP2A(PY1/PY2) genes. However, AGS and HEK293 expressing mutated LMP2A(Y74/85F) showed upregulated surface expression of HLA-ABC compared to vector control cells. Mutants were also screened for the expression of epigenetic regulators, DNMT1, DNMT3B, and UHRF1. Role of Y74/85F mutation within the ITAM region of LMP2A in the expression of epigenetic regulators at the transcript level of DNMT1, DNMT3B and UHRF1 was validated in AGS-LMP2A(PY1/PY2) and AGS-LMP2A(Y74/85F) cells. Decreased transcript-level expression of DNMT1, DNMT3B, and UHRF1 was observed in case of AGS-LMP2A(Y74/85F). AGS-LMP2A(PY1/PY2) exhibited similar expression pattern as the AGS-LMP2A cells (Supplementary Fig. 1D). Consistent with the previous results, immunoblotting analysis also showed that Y74 and Y85 in the ITAM region is responsible for the upregulated expression of epigenetic regulators (Fig. 2D). Increased methylated products of HLA-A, HLA-B, and HLA-C promoter regions were observed in AGS cells stably expressing LMP2A and LMP2A(PY1/PY2) genes through qRT-PCR (Fig. 2E). HEK293 cell transiently expressing LMP2A and LMP2A (PY1/PY2) displays increased protein level expression of DNMT1 (Supplementary Fig. 2A) and DNMT3B (Supplementary Fig. 2B) along with promoter hypermethylation of HLA-ABC gene (Supplementary Fig. 2D).

### Restoration of HLA-ABC expression upon treatment with 5′-azacytidine

**Figure 3:**
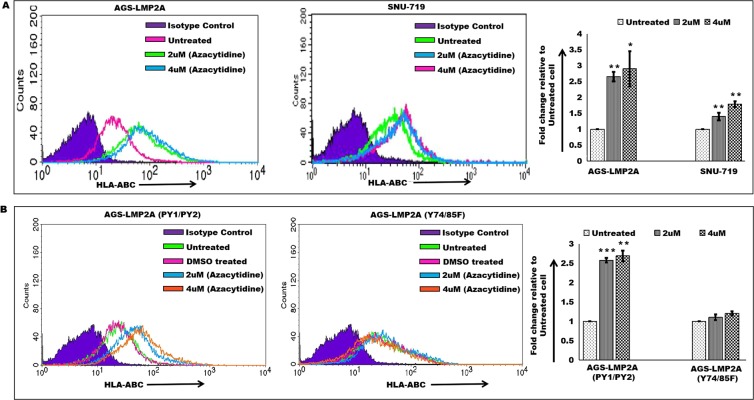
Restoration of HLA-ABC surface expression upon treatment with 5′-azacytidine. (**A**) HLA-ABC expression upon treatment with 5′-azacytidine in a dose (2uM and 4uM) dependent manner in AGS-LMP2A and SNU-719 cells relative to respective untreated cells. (**B**) HLA-ABC surface expression analysis upon treatment with 5′-azacytidine in a dose (2uM and 4uM) dependent manner in AGS-LMP2A (PY1/PY2) and AGS-LMP2A (Y74/85F) cells relative to respective untreated cells. Quantitation of results was performed by measuring median values using Cell Quest Pro software. Results are represented as mean ± s.e.m. of triplicate experiments. Data represent an average of n = 3 independent experiments. *P < 0.05, **P ≤ 0.01, ***P ≤ 0.001.

To further justify the role of methylation in downregulation of HLA Class Ia surface expression, LMP2A expressing epithelial cell carcinoma, SNU-719 and AGS-LMP2A were treated with 5′-azacytidine (removes methylation marks)^39^. Azacytidine treatment resulted in restoration of HLA-ABC surface expression in a dose-dependent manner in both AGS-LMP2A and SNU-719 cells (Fig. 3A). AGS-LMP2A(PY1/PY2) cells upon treatment with 5′-azacytidine displayed an increase in HLA-ABC surface expression in a dose-dependent manner (Fig. 3B). On the other hand, AGS-LMP2A(Y74/85F) cells upon treatment with 5′-azacytidine failed to show any significant increase in HLA-ABC surface expression in a dose-dependent manner (Fig. 3B). Thus, further certifying the role of the ITAM motif (Y74 and Y85) in downregulation of HLA-ABC surface expression through promoter hypermethylation.

### EBV-LMP2A regulates NKG2D ligands’ expression

**Figure 4:**
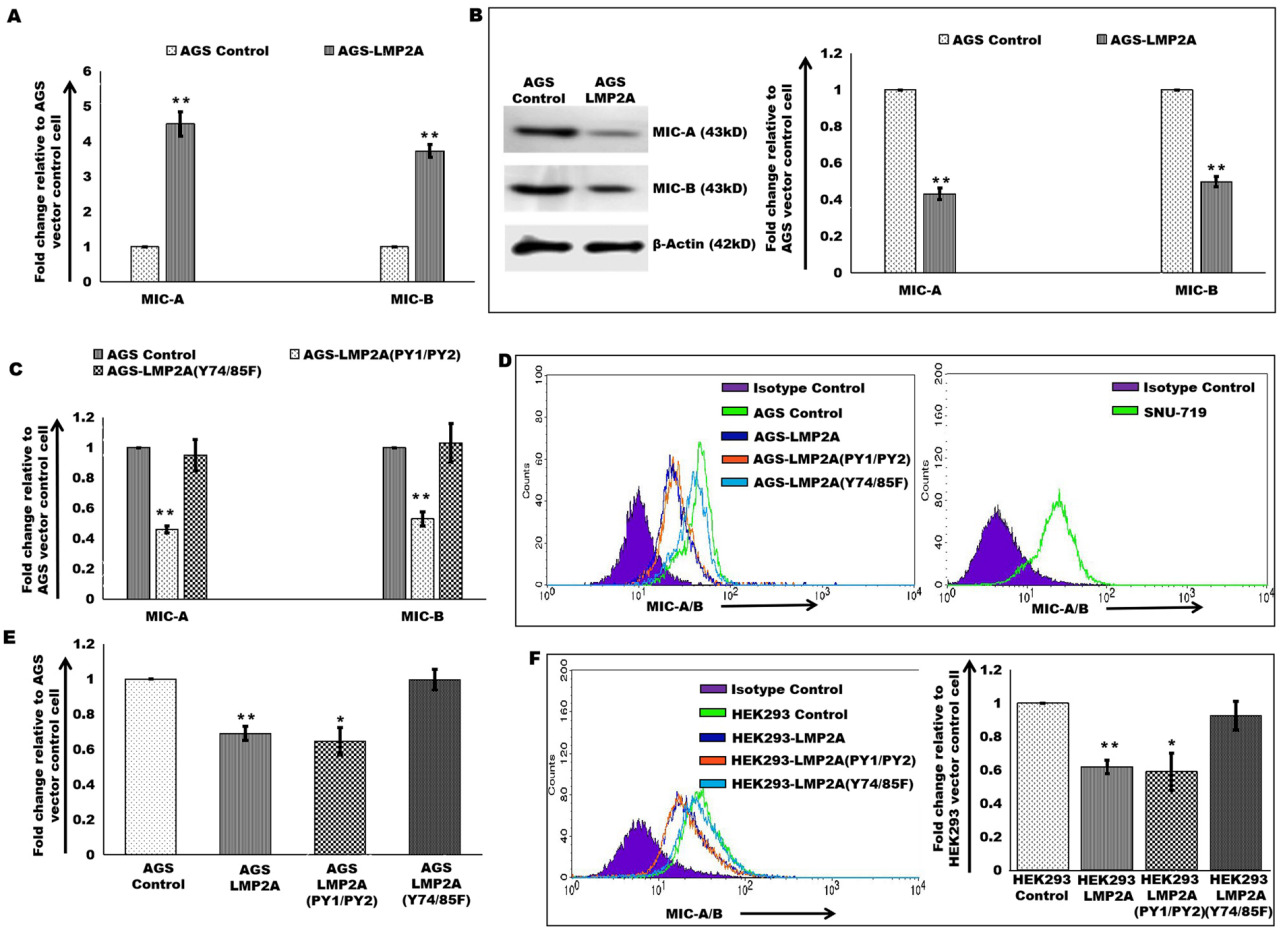
Expression of NKG2D ligands’, MIC-A/B. (**A**) qRT-PCR of NKG2D ligands’ (MIC-A and MIC-B) in AGS-LMP2A relative to AGS vector control cell. (**B**) Immunoblotting experiments to determine MIC-A and MIC-B levels in AGS vector control and AGS-LMP2A cells. (**C**) Protein level measurement of MIC-A and MIC-B in AGS-LMP2A (PY1/PY2) and AGS-LMP2A (Y74/85F) cells compared to AGS vector control cell. The bar graph depicts the densitometry quantification of relative expression levels. (**D** and **E**) MIC-A/B surface expression was determined in AGS-LMP2A, AGS-LMP2A (PY1/PY2) and AGS-LMP2A(Y74/85F) relative to AGS vector control cell. MIC-A/B surface expression was even studied for SNU-719 cells. (**F**) MIC-A/B surface expression determination upon introduction of LMP2A, LMP2A(PY1/PY2) and LMP2A (Y74/85F) into HEK293 cells relative to HEK293 vector control cell. Quantitation of results was performed by measuring median values using Cell Quest Pro software. Results are represented as mean ± s.e.m. of triplicate experiments. Data represent an average of n = 3 independent experiments. *P < 0.05, **P ≤ 0.01, ***P ≤ 0.001.

Major histocompatibility homologs, MIC-A/B acts as key regulators of NK cell-mediated cytotoxicity of virus-infected cells. Determination of the expression levels of MIC-A/B shows increased transcript-level expression in AGS LMP2A (Fig. 4A). Increased mRNA expression of MIC-A (Supplementary Fig. 4A) and MIC-B (Supplementary Fig. 4B) was also displayed in HepG2 cell expressing LMP2A and LMP2A(PY1/PY2) gene. Despite increased transcript level expression of MIC-A and MIC-B ligands’, a decreased expression pattern was encountered at translation level in association with LMP2A expression in AGS (Fig. 4B) and SNU5 (Supplementary Fig. 3A) cell. This decrease in protein-level expression in response to the LMP2A gene was further observed in AGS-LMP2A(PY1/PY2) (Fig. 4C). Consistent with the protein level expression, surface expression of MIC-A/B was also observed to be downregulated in AGS-LMP2A, AGS-LMP2A(PY1/PY2) and SNU-719 cells (Fig. 4D,E). Decreased MIC-A/B surface expression in response to viral oncoprotein, LMP2A was further validated in other epithelial cell carcinomas, HEK293 (Fig. 4F), SNU5 (Supplementary Fig. 3B) and HepG2 cells (Supplementary Fig. 4C). Transcript level expression of ULBPs (Supplementary Fig. 3E) along with expression levels of HLA-E, HLA-F, and HLA-G was also investigated in AGS-LMP2A cells (Supplementary Fig. 3E). Increased expression of NKG2D inhibitory ligand, HLA-E was detected in AGS cells expressing LMP2A gene.

### Increased expression of UPR protein markers

**Figure 5:**
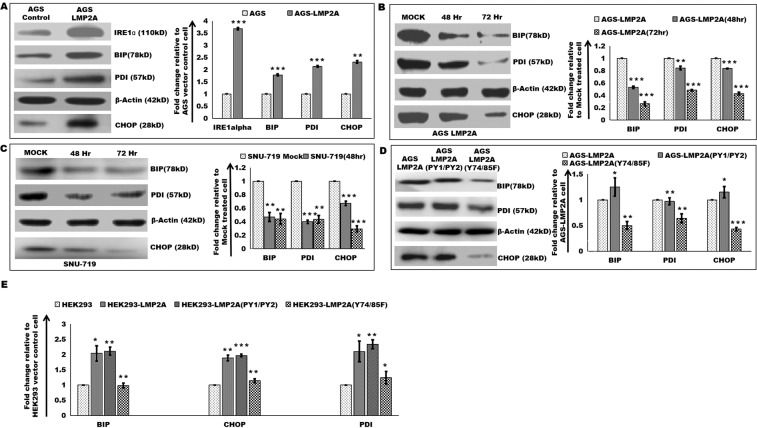
Increased expression of UPR protein markers. (**A**) Immunoblotting experiments to determine levels of UPR markers, iRE1-ɑ, BIP, PDI and CHOP in AGS vector control and AGS-LMP2A cells. The bar graph depicts the densitometry quantification of relative expression levels of iRE1-ɑ, BIP, PDI, and CHOP. (**B**) Immunoblotting experiments to determine BIP, PDI and CHOP levels upon siRNA mediated knockdown of LMP2A expression in AGS-LMP2A cells at 48 and 72 hr compared to Mock-treated cells. (**C**) Immunoblotting experiments to determine BIP, PDI and CHOP levels upon siRNA mediated knockdown of LMP2A expression in SNU-719 cells at 48 and 72 hr compared to Mock-treated cells. (**D**) Immunoblotting experiments to determine BIP, PDI and CHOP level in AGS-LMP2A (PY1/PY2) and AGS-LMP2A (Y74/85F) compared to AGS-LMP2A cells. (**E**) Protein level measurement of BIP, PDI and CHOP in HEK293 transiently expressing LMP2A, LMP2A(PY1/PY2) and LMP2A (Y74/85F) relative to HEK293 vector control cell. The bar graph depicts the densitometry quantification of relative expression levels. Results are represented as mean ± s.e.m. of triplicate experiments. Data represent an average of n = 3 independent experiments. *P < 0.05, **P ≤ 0.01, ***P ≤ 0.001.

NKG2D ligands’ are recognized to be regulated by a number of cellular stress, thus regulating the activation of NK cells^40–44^. UPR has recently been reported to regulate the expression of certain NKG2D ligands’^45^. Gastric cancer cell, expressing LMP2A (AGS-LMP2A) was analyzed for expression of UPR proteins, iRE1-ɑ, BIP, PDI, and CHOP. Immunoblotting analysis revealed increased expression of UPR proteins in AGS-LMP2A (Fig. 5A). EBV-negative gastric cancer cell, SNU5 transiently expressing LMP2A and LMP2A(PY1/PY2) also showed increased expression of PDI (Supplementary Fig. 3A). Decreased expression of UPR proteins was detected in AGS-LMP2A (Fig. 5B) and SNU-719 (Fig. 5C) cells upon time-dependent siRNA mediated knockdown of viral latent protein, LMP2A. Furthermore, decreased expression of UPR proteins was observed in AGS-LMP2A(Y74/85F), whereas AGS-LMP2A(PY1/PY2) showed similar expression pattern as AGS-LMP2A cells (Fig. 5D). UPR proteins were upregulated in response to LMP2A and LMP2A(PY1/PY2) by HEK293 cells (Fig. 5E).

### Restoration of MIC-A/B surface expression

**Figure 6:**
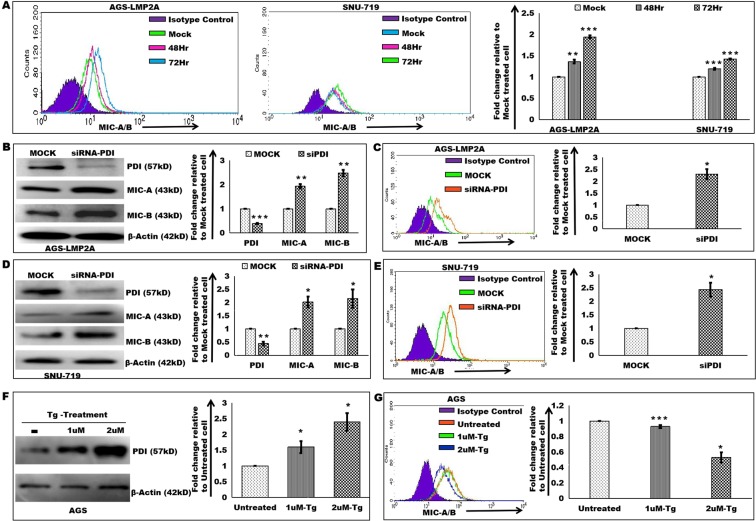
Restoration of NKG2D ligands’, MIC-A/B surface expression. (**A**) MIC-A/B surface expression determination upon siRNA mediated knockdown of LMP2A expression in AGS-LMP2A and SNU-719 cells relative to respective Mock treated cell. (**B**) PDI, MIC-A, MIC-B protein level determination upon knockdown of PDI in AGS-LMP2A cells compared to Mock treated cells. (**C**) MIC-A/B surface expression determination upon knockdown of PDI in AGS-LMP2A cells compared to Mock treated cells. (**D**) PDI, MIC-A, MIC-B protein level determination upon knockdown of PDI in SNU-719 cells compared to Mock treated cells. (**E**) MIC-A/B surface expression determination upon knockdown of PDI in SNU-719 cells compared to Mock treated cells. (**F**) Immunoblotting experiments to determine the expression of PDI in AGS cells upon thapsigargin (Tg) treatment in a dose (1uM and 2uM) dependent manner compared to the untreated cells. (**G**) MIC-A/B surface expression determination in AGS cells upon thapsigargin (Tg) treatment in a dose (1uM and 2uM) dependent manner compared to untreated cells. Quantitation of results was performed by measuring median values using Cell Quest Pro software. Results are represented as mean ± s.e.m. of triplicate experiments. Data represent an average of n = 3 independent experiments. *P < 0.05, **P ≤ 0.01, ***P ≤ 0.001.

Disulfide isomerases are known to regulate cancer migration through modulation of invasive properties^35^. PDI is a well-known UPR protein recognized to catalyze proteolytic shedding of cell surface proteins^33^. We sought to determine the role of PDI and viral oncoprotein, LMP2A in regulation of MIC-A/B surface expression. An increased surface level expression of MIC-A/B was observed in AGS-LMP2A and SNU-719 cells upon siRNA-mediated knockdown of LMP2A expression (Fig. 6A). Role of PDI in surface expression of MIC-A/B in LMP2A-expressing epithelial cell carcinoma was investigated through knockdown study of PDI, mediated by siRNA. MIC-A/B protein and surface-level expressions were restored in AGS-LMP2A cells (Fig. 6B,C) and SNU-719 cells (Fig. 6D,E). Knockdown analysis reveals the role of PDI in regulation of MIC-A/B surface expression through proteolytic shedding. AGS cells stably expressing the Y74/85F mutant version of LMP2A failed to show similar results as that of AGS-LMP2A cells. LMP2A ITAM region mutation (Y74/85F) was unable to exhibit similar phenotype as normal LMP2A gene. Previous reports show activation of PI3k-Akt pathway^15^ and Sonic Hedgehog (Shh) pathway^19^ by the viral latent protein, LMP2A. In order to investigate the role of these signaling pathways in regulation of MIC-A/B expression, we used Forskolin (PI3k/Akt inhibitor) and LY294002 (Shh inhibitor) in a dose-dependent manner. The surface expression of MIC-A/B was significantly enhanced in AGS-LMP2A cell (Supplementary Fig. 4D) and SNU-719 cell (Supplementary Fig. 4E) upon Forskolin treatment. These results suggest that decreased MIC-A/B surface expression in EBV infected epithelial cell carcinomas is due to activation of Shh pathway. Next, we further analyzed the role of UPR in MIC-A/B surface expression through thapsigargin (an UPR inducer drug) treatment. AGS (Fig. 6F,G) and SNU5 (Supplementary Fig. 3C,D) cells showed increased MIC-A/B surface expression in response to thapsigargin treatment in a dose (1uM and 2uM) dependent manner for a period of 6hr. Our results indicate regulation of MIC-A/B surface expression through PDI in LMP2A expressing epithelial cell carcinomas.

## Discussion

We previously reported EBV has developed strategies to avoid detection of infected cells by CTLs and compromising the host’s ability to overcome EBV infection in EBVaGC^17,18^. The current study addresses the role of EBV latent protein, LMP2A in the regulation of host immune response through epigenetic regulators and UPR in LMP2A-positive epithelial cell carcinomas. We first explored the role of epigenetics in the downregulation of HLA-ABC surface expression in response to viral oncogene, LMP2A. Furthermore, since alterations in epigenetic marks have recently been linked to the regulation of HLA class I gene expression^46^, we, therefore, proceeded to investigate the role of LMP2A in regulating the gene expression through epigenetic regulators. The promising aspect of epigenetic regulators in tumorigenesis has recently been investigated in several carcinomas^47^. Our results provide evidence for LMP2A mediated increased methylation of the HLA-ABC promoter region along with elevated levels of epigenetic regulators; DNMT1, DNMT3B, and UHRF-1. Hence it is significant that methylation mediated modification of HLA-ABC promoter region is utilized by the virus to target the availibility of HLA-ABC on the surface of infected cells. Methylation mediated downregulation of HLA-ABC expression was justified by treatment with 5′-azacytidine, which acts as a demethylating agent. An increased HLA-ABC surface expression was observed upon demethylation treatment in LMP2A-expressing gastric cancer cell, AGS and SNU-719. We also showed that LMP2A(Y74/85F) mutant failed to exhibit smiliar phenotype as LMP2A validating the role played by Tyr74 and Tyr85 in the downregulation of HLA-ABC expression.

**Figure 7:**
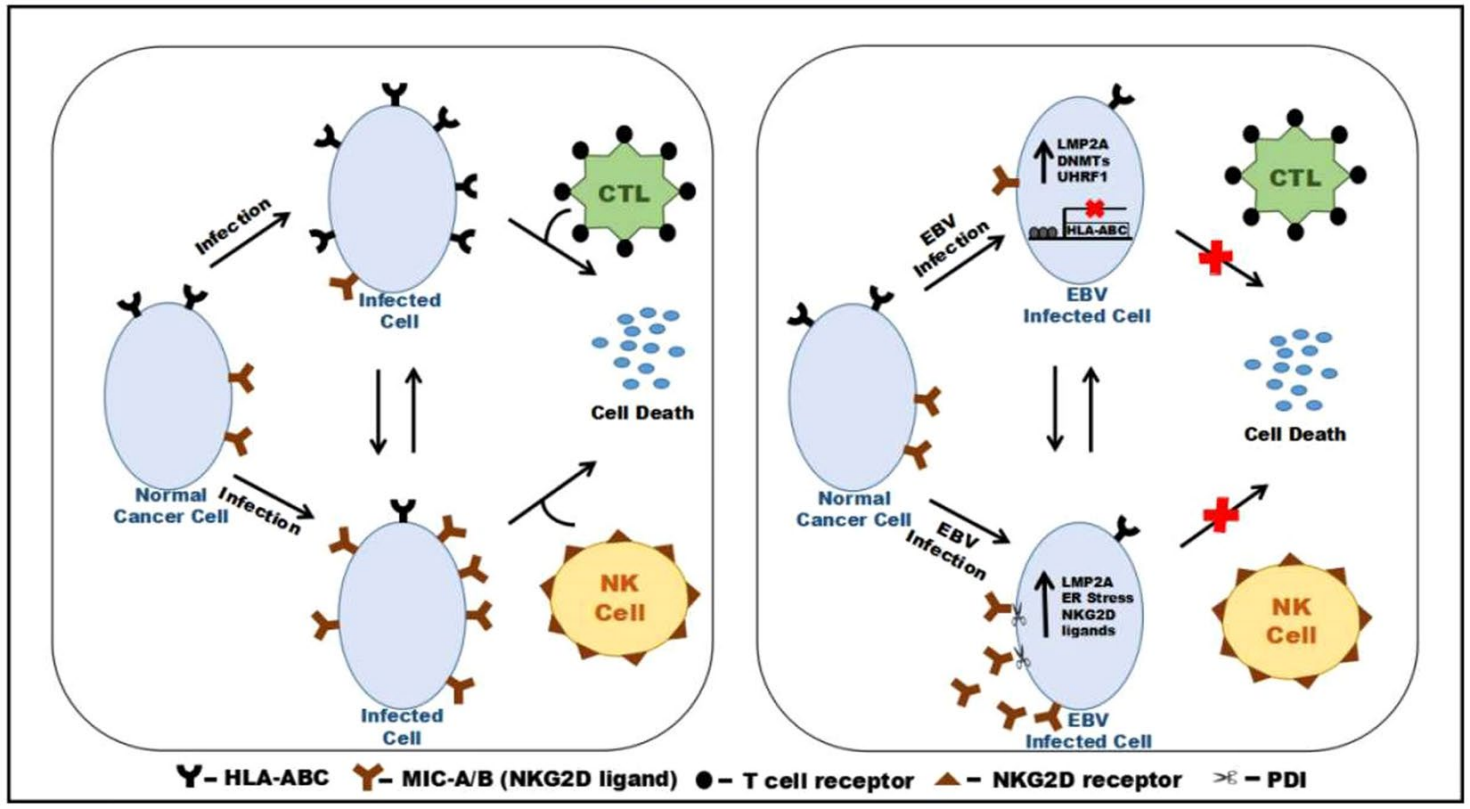
Schematic representation of the current findings. When normal epithelial cancer cell acquires a viral infection, infected cells are destined to cell death mediated by CTLs, where HLA-ABC plays an important role in presenting the viral antigenic peptides. If CTL response is downmodulated than infected cells must undergo lysis through NK cell response where NKG2D ligands on the surface of infected cells allow there recognition by the counteracting NK cells. In the presence of EBV-LMP2A, decreased expression of HLA-ABC is encountered on the surface of EBV infected cells through hypermethylation of the HLA-ABC promoter region. According to the ‘missing self’ hypothesis when there is downregulation of HLA class Ia expression, expression of NKG2D ligands’ must be upregulated such that the infected cells become prone to NK cell-mediated cytotoxicity. Interestingly, EBV-LMP2A is shown to target NKG2D ligands’ (MIC-A/B) expression on the surface of LMP2A-expressing epithelial cell carcinomas, extricating them from NK cell-mediated lysis.

Apart from CTL response, NK cell response provides protection against viral infection. NKG2D ligands’ expression on the infected cells play important role in recognition of virus-infected cells by the counteracting NK cell. Expression of NKG2D ligands’ is transcriptionally regulated by various types of cellular stresses associated with transformation, viral infection, or other stress signals to the host cell. The stress signals that alerts the immune cells includes DNA damage response^42^, oxidative stress^40^, and UPR^45^ during cancer progression. In an earlier study, EBV was shown to downregulate MIC-B through BARTs^27^. LMP2A-deficient EBV infected lymphoblastoid cell lines (LCLs) showed increased susceptibility to CTLs^48^. HLA-E expression also plays important role in desensitization of NK cells^49^. UPR proteins acts as an important regulator for viral persistence and function. Our current findings were in good agreement with EBV latent protein, LMP2A mediated loss of MIC-A/B surface expression. Meanwhile expression of UPR proteins was also altered in LMP2A expressing epithelial cell carcinomas. It might be suggested that these expression alterations of UPR proteins might result in regulation of MIC-A/B expression. PDI is localized on the plasma membrane as well as within the cytoplasm, where it is reported to facilitate proteolytic shedding of cell surface proteins^35^. We showed that PDI resulted in regulation of MIC-A/B surface expression in LMP2A-expressing epithelial cell carcinomas. Thus, PDI could be used as drug target to prevent tumour immune evasion. We verified the role of ITAM (Tyr74 and Tyr85) in the downregulated expression of MIC-A/B mediated by PDI. Replacement of LMP2A with LMP2A ITAM mutant (Y74/85F) was successful in releasing the effect viral protein on the surface availibility of MIC-A/B in epithelial cell carcinomas. ITAM region is reported to activate several cellular signaling cascades including PI3k-Akt and Shh pathways. Thus the two pathways were targeted using LY294002 and Forskolin, respectively. Restoration of MIC-A/B surface expression was observed in response to Forskolin treatment, suggesting the role of the Shh pathway in the regulation of MIC-A/B surface expression. The current study highlights, regulation of CTL response through decreased HLA-ABC expression along with modulation of NK cell response through downregulation of MIC-A/B surface expression in LMP2A expressing epithelial cell carcinomas (Fig. 7).

In summary, EBV-LMP2A acts as a potent regulator of host immune response. Evasion of immune response acts as a marker for poor prognosis in cancer patients, particularly in epithelial carcinomas. These regulation of immune response through promoter hypermethylation and UPR proteins provides better understanding about the strategies utilised by EBV to overcome recognition by immune cells.

## Materials and methods

Materials. Thapsigargin, 5′-azacytidine, Forskolin, along with LY294002 were purchased from Sigma Aldrich and dissolved in DMSO for experimental treatment, while G418 was purchased from Gibco and solubilized in PBS for the selection of clones stably expressing pcDNA-3.1 along with the gene of interest. The HLA-ABC and MIC-A/B antibody was procured from BD Biosciences. Antibodies against DNMT1, DNMT3B, UHRF1, MIC-A, MIC-B, LMP2A, and β-ACTIN were purchased from Abcam and BIP, CHOP, IRE1 ɑ and PDI primary antibodies, along with anti-rabbit, anti-mouse, anti-rat horseradish peroxidase (HRP)-conjugated secondary antibodies were procured from Cell Signaling Technology. Antibodies’ information is provided in Supplementary Table 3.

Cell Culture. Human gastric cancer cell lines, AGS (EBV-negative) and SNU-719 (EBV-positive)^12,18,19^ were cultured in RPMI 1640 (Gibco) supplemented with 10% (v/v) heat-inactivated fetal bovine serum, FBS (Gibco). Human Embryonic Kidney 293 (HEK293, EBV-negative)^19^, human hepatocellular carcinoma (HepG2, EBV-negative) cells were grown in DMEM supplemented with 10%(v/v) FBS. Human gastric cancer cells, SNU5 (EBV-negative) were grown in DMEM (Gibco) supplemented with 20%(v/v) FBS. Cell lines were maintained with 5% CO_2_ humidified atmosphere at 37 °C.

DNA constructs and transfection. LMP2A cDNA and LMP2A ITAM mutants were gifted by Prof. R. Longnecker (Northwestern University, Chicago, USA). LMP2A cDNA along with LMP2A ITAM mutant (PY1/PY2 and Y74/85F) cDNA were cloned into the vector pcDNA3.1 (Invitrogen) to obtain the LMP2A/pcDNA, LMP2A (PY1/PY2)/pcDNA and LMP2A (Y74/85F)/pcDNA respectively. The plasmids were delivered into the EBV-negative cell line, AGS through Lipofectamine 2000 (Invitrogen). Stable clones of transfectants expressing only vector pcDNA, LMP2A, and mutant LMP2A were selected in medium containing 800 mg/mL of G418 (Gibco). LMP2A and mutant versions of LMP2A were transiently transfected in HEK293, HepG2 and SNU5 cells utilizing lipofectamine 2000.

RT-PCR and quantitative real-time PCR. RNA was extracted through Tripure isolation reagent (TRIZOL, Roche) and quality assayed using spectroscopy (Eppendorf BioPhotometer). 2ug of the total RNA was subjected to reverse transcription for 90 min at 42 °C using oligo dT primers and M-MuLV reverse transcriptase (Fermentas). Quantitative RT-PCR was performed using SYBR Green core PCR reagents (Applied Biosystems) and HPRT was used as the endogenous control. The qRT-PCR reactions and analysis were carried out in the 7500 Sequence Detection System (Applied Biosystems)^12,19^. Primer sequences used for qRT-PCR analysis in this study are provided in Supplementary Table 1.

Bisulfite Modification and Methylation Specific PCR (MSP). 1 × 10^5^ cells were harvested and dissolved in 100 mL 5% PBS was used for further experiment. EpiTect Fast LyseAll Bisulfite Kit (Qiagen) was used to carry out bisulfite modification as per the manufacturer’s instructions. The modified DNA samples were finally stored at −20 °C until further use. The methylation status of the cell lines was determined using MSP. Primer sequences used for MSP in this study are shown in Supplementary Table 2. Polymerase chain reaction (PCR) was carried out in a volume of 50 μl high GC buffer with 100 ng or less template DNA with TaKaRa EpiTaq HS (TaKaRa). There were 35 cycles of denaturation at 98 °C for 10 s, annealing at 60 °C for 30 s, and extension at 72 °C. The amplified product was assayed using DNA gel electrophoresis and photographed using a Gel Doc chemiluminescence detector (BioRad). The qRT-PCR reaction was performed using SYBR Green core PCR reagents and bisulfite modified genomic DNA as a template and methylated GAPDH was used as the endogenous control. Primer sequences used for qRT-PCR for methylation study are provided in Supplementary Table 2.

Azacytidine treatment. The cell lines were plated at low density, incubated with 2uM and 4uM 5′-Aza-2′-deoxycytidine for 6 days, independent sets were replenished with fresh 5′-Aza-2′-deoxycytidine every 24 hr. The cells were harvested after treatment for surface expression of HLA-ABC.

Western blot analysis. Cells were harvested and lysed utilizing Nonidet P-40-lysis buffer [20 mM Tris-HCl (pH 8.0), 137 mM NaCl, 10% glycerol, 1% Nonidet P-40, 2 mM EDTA, 200 mM Na_3_VO_4,_ 100 mM phenylmethylsulfonyl fluoride, protease inhibitor cocktail (Roche) and phosphatase inhibitor (Roche, Mannheim, Germany)] for 30 min on ice, further centrifuged at 16,000 g for 5 min at 4 °C and the supernatant was collected for further analysis. Equal amounts of protein (25–50 mg) were subjected to 10–12% SDS-polyacrylamide gel electrophoresis and electrotransferred onto a nitrocellulose membrane (GE Healthcare). The membrane was subjected to blocking with 5% BSA in TBS containing 0.1% Tween 20 (Sigma Chemical Co.) for an hour at room temperature. Primary antibodies mentioned previously were incubated with membranes at 4 °C overnight. Membranes were then washed in TBS-T, probed with secondary antibodies conjugated to horseradish peroxidase (Cell Signaling Technology) for an hour (1:3000 dilution in TBS-T), and then washed with TBS-T. The antibody-bound protein bands were detected through enhanced chemiluminescence reagent (Amersham Biosciences) and photographed using a VersaDoc chemiluminescence detector and Quantity ONE software (BioRad)^12,19^.

RNA interference. The siRNA sequence for inhibiting LMP2A mRNA expression was 5′-AACUCCCAAUAUCCAUCUGCU-3′, identical to that previously reported^12,19,50^. The siRNA sequence for inhibiting PDI was 5′-GGACCAUGAGAACAUCGUC-3′ similar to that previously reported^51^. A control scrambled sequence was also used as Mock (Sigma Aldrich, St. Louis, MO). The siRNAs were delivered utilizing Lipofectamine 2000 (Invitrogen) and the cells were further assayed at different time intervals.

Cell surface analysis of HLA-ABC. 1 × 10^5^ cells were dissolved in 100 uL 5% PBS and incubated with allophycocyanin (APC) conjugated anti-HLA-ABC monoclonal antibody for a time period of 30 min in dark at 4 °C. The amount of antibody used was as per the manufacturer’s instructions. Stained samples were then subjected to PBS wash, further resuspended in 1 ml 5% PBS and endured to Fluorescence-Activated Cell Sorting (FACS) Analysis through FACS Calibur (Becton Dickinson). The results were analyzed using Cell Quest Pro software (B.D. Biosciences)^19^.

Cell surface analysis of MIC-A/B. 1 × 10^5^ cells were dissolved in 100 uL 5% PBS and incubated with phycoerythrin (PE) conjugated anti-MIC-A/B monoclonal antibody for a time period of 30 min in dark at 4 °C. The amount of antibody used was as per the manufacturer’s instructions. Stained samples were then subjected to PBS wash, further resuspended in 1 ml 5% PBS and endured to Fluorescence-Activated Cell Sorting (FACS) Analysis through FACS Calibur (Becton Dickinson). The results were analyzed using the Cell Quest Pro software (B.D. Biosciences).

Thapsigargin (Tg) treatment. The cell lines were plated at low density, incubated with 1uM and 2uM thapsigargin for a time period of 6 hr. The cells were then harvested for protein level expression of PDI and surface expression of MIC-A/B.

Statistical analysis. Results obtained from all the experiments were reported as the mean ± s.e.m. The significance of the analyzed data was calculated by a standard student’s t-test. The gene expression levels were compared by unpaired two-tailed t-test. Only P < 0.05 was considered statistically significant.

## Supplementary information

Supplementary Information

## References

[1] Hicklin DJ, Marincola FM, Ferrone S (1999). HLA class I antigen downregulation in human cancers: T-cell immunotherapy revives an old story. Mol. Med. Today.

[2] Restifo NP (1993). Identification of human cancers deficient in antigen processing. J Exp Med.

[3] Vitale M (1998). HLA class I antigen and transporter associated with antigen processing (TAP1 and TAP2) down-regulation in high-grade primary breast carcinoma lesions. Cancer Res..

[4] Groh V, Wu J, Yee C, Spies T (2002). Tumour-derived soluble MIC ligands impair expression of NKG2D and T-cell activation. Nature..

[5] Pende D (2002). Major histocompatibility complex class I-related chain A and UL16-binding protein expression on tumour cell lines of different histotypes. Cancer Res..

[6] Thompson MP, Kurzrock R (2004). Epstein-Barr Virus and Cancer. Clin Cancer Res..

[7] Longnecker, R. M., Kieff, E. & Cohen, J. I. Epstein-Barr virus/replication and Epstein-Barr virus, In Virology, Fields, 6th ed.; Knipe, D. M., Howley, P. M. Eds.; Lippincott, Williams and Wilkins: Philadelphia, PA, USA, 1898–1979 (2013).

[8] Epstein MA, Achong BG, Barr YM (1964). Virus particles in cultured lymphoblasts from Burkitt’s lymphoma. Lancet.

[9] Elgui de Oliveira D, Müller-Coan BG, Pagano JS (2016). Viral Carcinogenesis Beyond Malignant Transformation: EBV in the Progression of Human Cancers. Trends Microbiol..

[10] Takada, K. *et al*. *Epstein-Barr virus and gastric carcinoma*. 255–261 (2000). 10.1136/mp.53.5.255 PMC1186978 11091849

[11] Scholle F, Bendt KM (2000). Epstein-Barr Virus LMP2A Transforms Epithelial Cells, Inhibits Cell Differentiation, and Activates Akt. J. Virol..

[12] Pal Anindita Deb, Basak Nandini Pal, Banerjee Aditi Sengupta, Banerjee Subrata (2014). Epstein–Barr virus latent membrane protein-2A alters mitochondrial dynamics promoting cellular migration mediated by Notch signaling pathway. Carcinogenesis.

[13] Fotheringham JA, Coalson NE, Raab-traub N (2012). Epstein-Barr Virus Latent Membrane Protein-2A Induces ITAM/Syk- and Akt-Dependent Epithelial Migration through ɑV-Integrin Membrane Translocation. J. Virol..

[14] Morrison JA, Raab-traub N, Carolina N, Hill C, Carolina N (2005). Roles of the ITAM and PY Motifs of Epstein-Barr Virus Latent Membrane Protein 2A in the Inhibition of Epithelial Cell Differentiation and Activation of β-Catenin Signaling. J. Virol..

[15] Fukuda M, Longnecker R (2007). Epstein-Barr Virus Latent Membrane Protein 2A Mediates Transformation through Constitutive Activation of the Ras/PI3-K/Akt Pathway. J. Virol..

[16] Frisan Teresa, Zhang Qian-Jin, Levitskaya Jelena, Coram Michael, Kurilla Michael G., Masucci Maria G. (1996). Defective presentation of MHC class I-restricted cytotoxic T-cell epitopes in Burkitt's lymphoma cells. International Journal of Cancer.

[17] Shen Lijun, Chiang Alan K.S., Liu Wei Ping, Li Gan Di, Liang Raymond H.S., Srivastava Gopesh (2001). Expression of HLA class I, ?2-microglobulin, TAP1 and IL-10 in Epstein-Barr virus-associated nasal NK/T-cell lymphoma: Implications for tumor immune escape mechanism. International Journal of Cancer.

[18] Dutta Nirmal, Gupta Arnab, Mazumder Debendra Nath Guha, Banerjee Subrata (2006). Down-regulation of locus-specific human lymphocyte antigen class I expression in Epstein–Barr virus-associated gastric cancer. Cancer.

[19] Pal AD, Banerjee S (2015). Epstein-Barr virus latent membrane protein 2A mediated activation of Sonic Hedgehog pathway induces HLA class Ia downregulation in gastric cancer cells. Virology..

[20] Baylin SB, Herman JG (2000). DNA hypermethylation in tumourigenesis epigenetics joins genetics. Trends Genet..

[21] Zhou L (2015). Uhrf1 promotes proliferation of gastric cancer via mediating tumour suppressor gene hypermethylation. Cancer Biol. Ther..

[22] Ljunggren H-G, Karre K (1990). In search of the ‘missing self’: MHC molecules and NK cell recognition. Immunol. Today..

[23] Guerra Nadia, Tan Ying Xim, Joncker Nathalie T., Choy Augustine, Gallardo Fermin, Xiong Na, Knoblaugh Susan, Cado Dragana, Greenberg Norman R., Raulet David H. (2008). NKG2D-Deficient Mice Are Defective in Tumor Surveillance in Models of Spontaneous Malignancy. Immunity.

[24] McGilvray R. W., Eagle R. A., Watson N. F.S., Al-Attar A., Ball G., Jafferji I., Trowsdale J., Durrant L. G. (2009). NKG2D Ligand Expression in Human Colorectal Cancer Reveals Associations with Prognosis and Evidence for Immunoediting. Clinical Cancer Research.

[25] Matusali G (2013). Soluble ligands for the NKG2D receptor are released during HIV-1 infection and impair NKG2D expression and cytotoxicity of NK cells. FASEB J..

[26] Waldhauer Inja, Steinle Alexander (2006). Proteolytic Release of Soluble UL16-Binding Protein 2 from Tumor Cells. Cancer Research.

[27] Komabayashi Yuki, Kishibe Kan, Nagato Toshihiro, Ueda Seigo, Takahara Miki, Harabuchi Yasuaki (2016). Circulating Epstein-Barr virus-encoded micro-RNAs as potential biomarkers for nasal natural killer/T-cell lymphoma. Hematological Oncology.

[28] Shen X, Zhang K, Kaufman RJ (2004). The unfolded protein response — a stress signaling pathway of the endoplasmic reticulum. J. Chem. Neuroanat..

[29] Schroder M, Kaufman RJ (2005). ER stress and the unfolded protein response. Mutat. Res..

[30] Hsiao JR (2009). Endoplasmic reticulum stress triggers XBP-1-mediated up-regulation of an EBV oncoprotein in nasopharyngeal carcinoma. Cancer Res..

[31] Kaiser Brett K., Yim Daesong, Chow I-Ting, Gonzalez Segundo, Dai Zhenpeng, Mann Henning H., Strong Roland K., Groh Veronika, Spies Thomas (2007). Disulphide-isomerase-enabled shedding of tumour-associated NKG2D ligands. Nature.

[32] Essex DW, Li M, Miller A, Feinman RD (2001). Protein disulfide isomerase and sulfhydryl-dependent pathways in platelet activation. Biochemistry.

[33] Ellgaard L, Ruddock LW (2005). The human protein disulphide isomerase family: substrate interactions and functional properties. EMBO Rep.,.

[34] Turano C, Coppari S, Altieri F, Ferraro A (2002). Proteins of the PDI family: unpredicted non-ER locations and functions. J Cell Physiol..

[35] Goplen. D (2006). Protein disulfide isomerase expression is related to the invasive properties of malignant glioma. Cancer Res.,.

[36] Birdwell CE (2014). Genome-Wide DNA Methylation as an Epigenetic Consequence of Epstein-Barr Virus Infection of Immortalized Keratinocytes. J. Virol..

[37] Johnson DR (2003). Locus-specific constitutive and cytokine induced HLA class I gene expression. J. Immunol..

[38] Herman JG, Graff JR, Myohanen S, Nelkin BD, Baylin SB (1996). Methylation-specific PCR: A novel PCR assay for methylation status of CpG islands. Proc Natl Acad Sci USA.

[39] Serrano AS (2001). Expression of HLA class I antigens and restoration of antigen-specific ctl response in melanoma cells following 5-aza-2′-deoxycytidine treatment. Int. J. Cancer..

[40] Raulet DH, Marcus A, Coscoy L (2017). Dysregulated cellular functions and cell stress pathways provide critical cues for activating and targeting natural killer cells to transformed and infected cells. Immunol Rev..

[41] Raulet DH, Gasser S, Gowen BG, Deng W, Jung H (2014). Regulation of ligands for the NKG2D activating receptor. Annu. Rev. Immunol..

[42] Gasser Stephan, Orsulic Sandra, Brown Eric J., Raulet David H. (2005). The DNA damage pathway regulates innate immune system ligands of the NKG2D receptor. Nature.

[43] Venkataraman Gopalakrishnan M., Suciu Dominic, Groh Veronika, Boss Jeremy M., Spies Thomas (2007). Promoter Region Architecture and Transcriptional Regulation of the Genes for the MHC Class I-Related Chain A and B Ligands of NKG2D. The Journal of Immunology.

[44] Gowen, B. G. *et al*. A forward genetic screen reveals novel independent regulators of ULBP1, an activating ligand for natural killer cells. *Elife*, 10.7554/eLife.08474 (2015). 10.7554/eLife.08474 PMC4629278 26565589

[45] Hosomi S (2017). Intestinal epithelial cell endoplasmic reticulum stress promotes MULT1 up-regulation and NKG2D-mediated inflammation. J. Exp. Med..

[46] Nie Y (2001). DNA hypermethylation is a mechanism for loss of expression of the HLA class I genes in human esophageal squamous cell carcinomas. Carcinogenesis..

[47] Esteller M (2005). Aberrant DNA methylation as a cancer-inducing mechanism. Annu Rev Pharmacol. Toxicol..

[48] Rancan Chiara, Schirrmann Leah, Hüls Corinna, Zeidler Reinhard, Moosmann Andreas (2015). Latent Membrane Protein LMP2A Impairs Recognition of EBV-Infected Cells by CD8+ T Cells. PLOS Pathogens.

[49] Wischhusen J, Friese MA, Mittelbronn M, Meyermann R, Weller M (2005). HLA-E protects glioma cells from NKG2D-mediated immune responses *in vitro*: Implications for immune escape *in vivo*. J. Neuropathol. Exp. Neurol..

[50] Shin JY, Kim JO, Lee SK, Chae HS, Kang JH (2010). LY294002 may overcome 5-FU resistance via down-regulation of activated p-Akt in Epstein–Barr viruspositive gastric cancer cells. BMC Cancer.

[51] Schlotawa L (2018). Recognition and ER quality control of misfolded formylglycine-generating enzyme by protein disulfide isomerase. Cell Rep..

