# Supplementary material for: Downregulation of HLA-ABC expression through promoter hypermethylation and downmodulation of MIC-A/B surface expression in LMP2A-positive epithelial carcinoma cell lines
Source: Sci Rep. 2020 Mar 25;10:5415. doi: 10.1038/s41598-020-62081-0 (PMC7096436; doi:10.1038/s41598-020-62081-0)
Supplement: Supplementary file 1 — Supplementary Information [file 41598_2020_62081_MOESM1_ESM.docx]

**Downregulation of HLA-ABC expression through promoter hypermethylation and downmodulation of MIC-A/B surface expression in LMP2A-positive epithelial carcinoma cell lines**

Shweta Singh^1^ and Subrata Banerjee^1*^

^1^Biophysics and Structural Genomics Division, Saha Institute of Nuclear Physics, Homi Bhabha National Institute, 1/AF Bidhannagar, Kolkata, 700064, India

*To whom correspondence should be addressed. Tel: +91 33 23370379; Fax: +91 33 23374637; Email: [subrata.banerjee@saha.ac.in](mailto:subrata.banerjee@saha.ac.in)


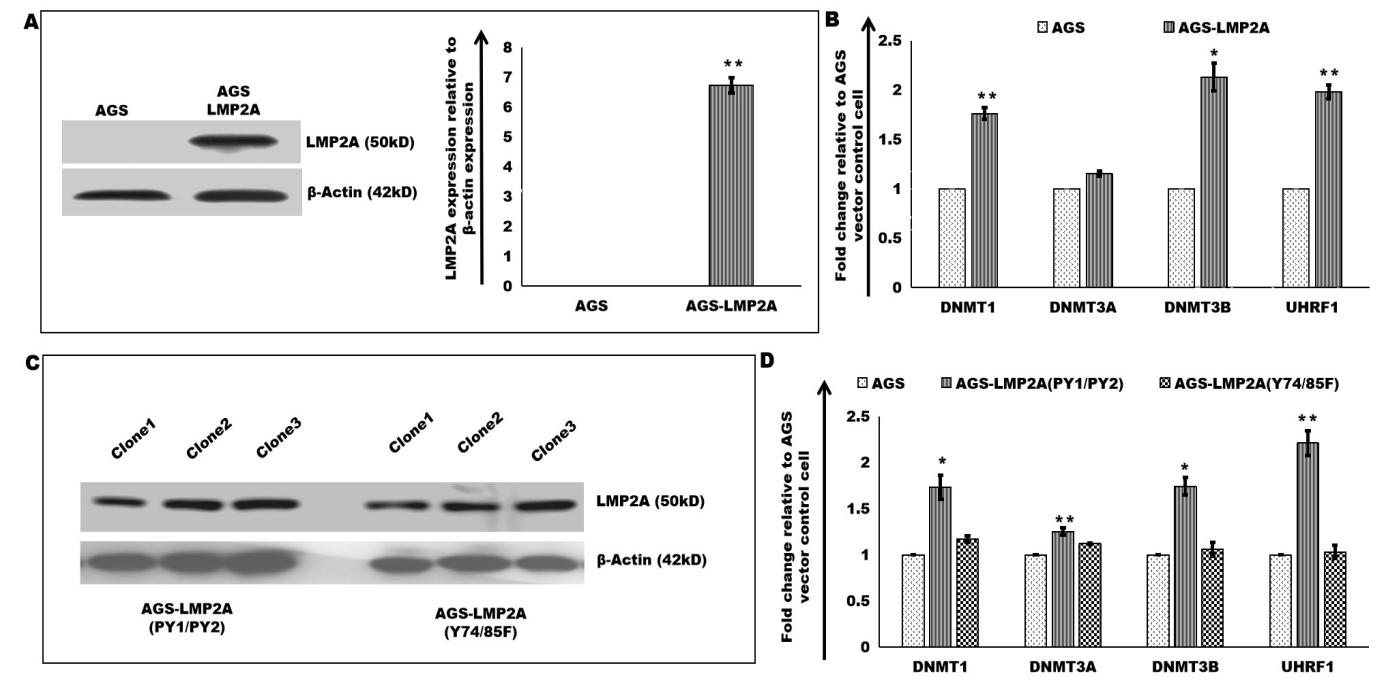


Supplementary Figure 1. (A) Immunoblotting analysis of LMP2A in AGS and AGS-LMP2A cells. Bar graph depicts the densitometry quantiﬁcation of relative expression of LMP2A in AGS and AGS-LMP2A cells compared to respective β-Actin expression. (B) qRT-PCR of epigenetic regulators (DNMTs and UHRF1) in AGS-LMP2A cells relative to AGS vector control cell. (C) Immunoblotting analysis of LMP2A in AGS cells stably expressing the mutant LMP2A genes (PY1/PY2 and Y74/85F). (D) qRT-PCR of epigenetic regulators (DNMTs and UHRF1) in AGS-LMP2A (PY1/PY2) and AGS-LMP2A(Y74/85F) cells relative to AGS vector control cell. Results are represented as mean ±s.e.m. of triplicate experiments. Data represents an average of n=3 independent experiments.*P < 0.05, **P ≤ 0.01, ***P ≤ 0.001.


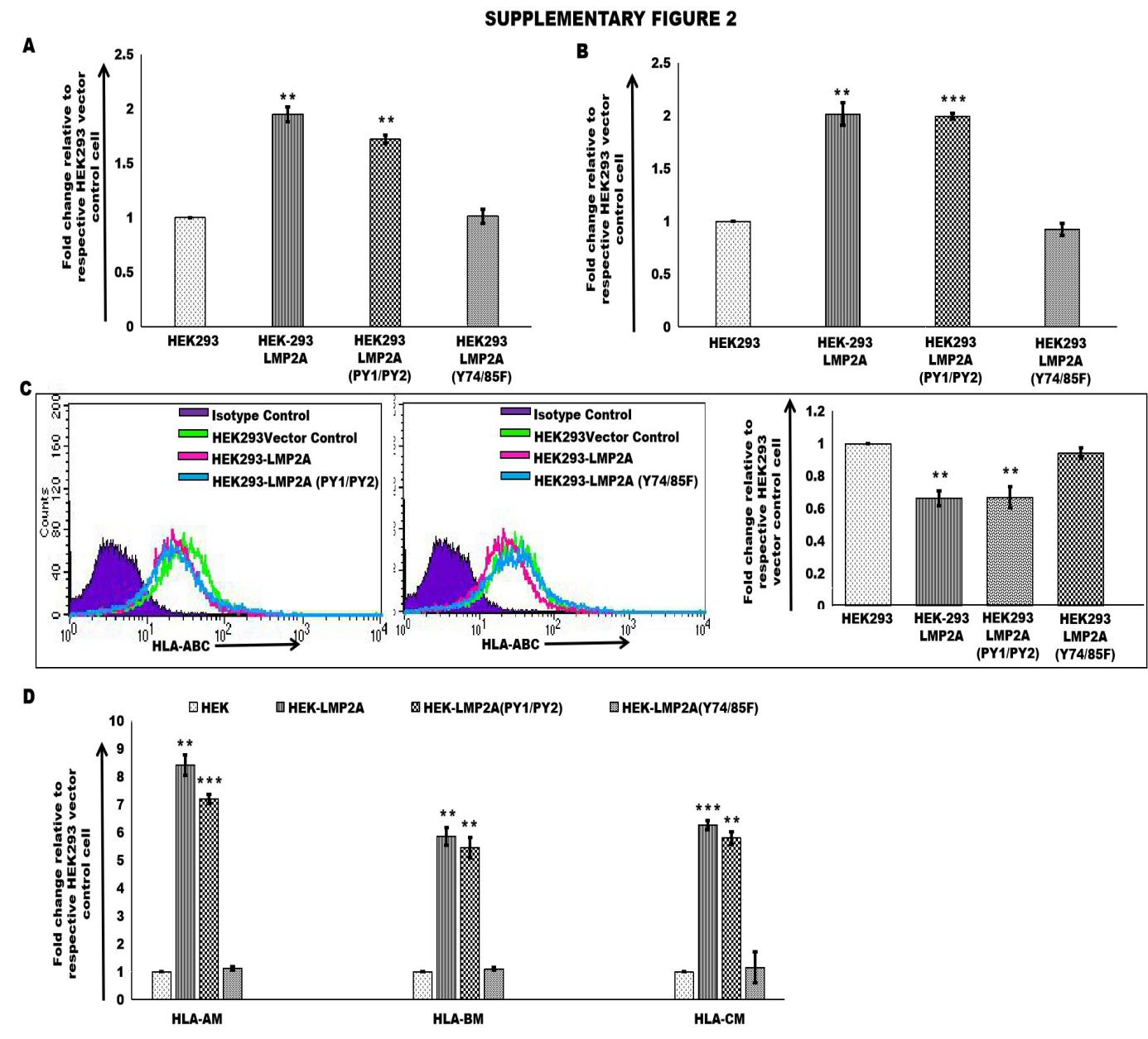


Supplementary Figure 2. (A) Protein level measurement of DNMT1 in HEK293, HEK293-LMP2A, HEK293-LMP2A(PY1/PY2) and HEK293-LMP2A(Y74/85F) cells compared to HEK293 vector control cells. (B) Protein level measurement of DNMT3B in HEK293, HEK293-LMP2A, HEK293-LMP2A(PY1/PY2) and HEK293-LMP2A(Y74/85F) cells compared to HEK293 vector control cells. (C) HLA-ABC surface expression analysis upon introduction of mutant LMP2A (PY1/PY2) and LMP2A(Y74/85F) into HEK293 cells relative to HEK293 vector control cell. Quantitation of results was performed by measuring median values through Cell Quest Pro software. (D) qRT-PCR of bisulfite modified genomic DNA template using different set of primers specific for methylation status of HLA-A, HLA-B and HLA-C promoter region in HEK293, HEK293-LMP2A, HEK293-LMP2A(PY1/PY2) and HEK293-LMP2A(Y74/85F) cells. Results are represented as mean ±s.e.m. of triplicate experiments. Data represents an average of n=3 independent experiments.*P < 0.05, **P ≤ 0.01, ***P ≤ 0.001.


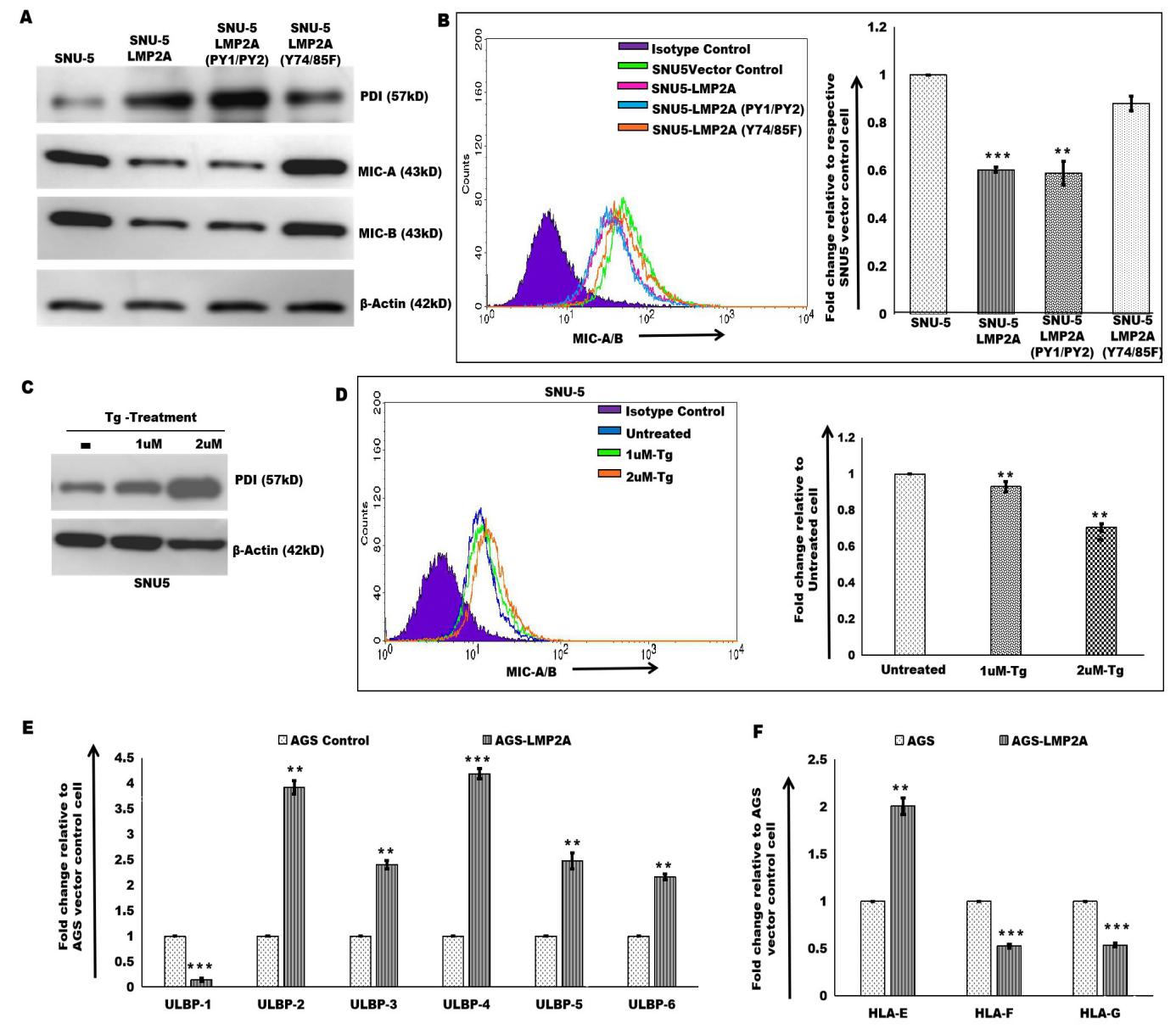


Supplementary Figure 3. (A) Immunoblotting analysis of PDI, MIC-A and MIC-B in SNU5, SNU5-LMP2A, SNU5-LMP2A(PY1/PY2) and SNU5-LMP2A(Y74/85F). (B) MIC-A/B surface expression analysis upon introduction of LMP2A, mutant LMP2A (PY1/PY2) and LMP2A(Y74/85F) into SNU5 cells relative to SNU5 vector control cell. Quantitation of results was performed by measuring median values using Cell Quest Pro software. (C) Immunoblotting experiments to determine level of PDI in SNU5 cells upon thapsigargin (Tg) treatment in a dose (1uM and 2uM) dependent manner compared to untreated cells. (D) MIC-A/B surface expression determination in SNU5 cells upon thapsigargin (Tg) treatment in a dose (1uM and 2uM) dependent manner compared to untreated cells. (E) qRT-PCR to analyze ULBP(1-6) transcript level expression in AGS-LMP2A cells relative to AGS vector control cell. (F) qRT-PCR to analyze HLA-E, HLA-F and HLA-G transcript level expression in AGS-LMP2A cells relative to AGS vector control cell. Quantitation of results was performed by measuring median values using Cell Quest Pro software. Results are represented as mean ±s.e.m. of triplicate experiments. Data represents an average of n=3 independent experiments.*P < 0.05, **P ≤ 0.01, ***P ≤ 0.001.

Supplementary Figure 4. (A) Quantitative RT-PCR to analyze MIC-A transcript level expression in HepG2-LMP2A, HepG2-LMP2A(PY1/PY2) and HepG2-LMP2A(Y74/85F) cells relative to HepG2 vector control cell. (B) Quantitative RT-PCR to analyze MIC-B transcript level expression in HepG2-LMP2A, HepG2-LMP2A(PY1/PY2) and HepG2-LMP2A(Y74/85F) cells relative to HepG2 vector control cell. (C) MIC-A/B surface expression upon introduction of mutant LMP2A(PY1/PY2) and LMP2A(Y74/85F) into HepG2 cells relative to HepG2 control cell. Quantitation of results was performed by measuring median values using Cell Quest Pro software. (D) MIC-A/B surface expression determination upon Forskolin and LY294002 treatment in a dose dependent manner in AGS-LMP2A cells compared to untreated cells. (H) MIC-A/B surface expression determination upon Forskolin and LY294002 treatment in a dose dependent manner in SNU-719 cells compared to untreated cells. Quantitation of results was performed by measuring median values using Cell Quest Pro software. Results are represented as mean ±s.e.m. of triplicate experiments. Data represents an average of n=3 independent experiments.*P ≤ 0.05, **P ≤ 0.01, ***P ≤ 0.001.


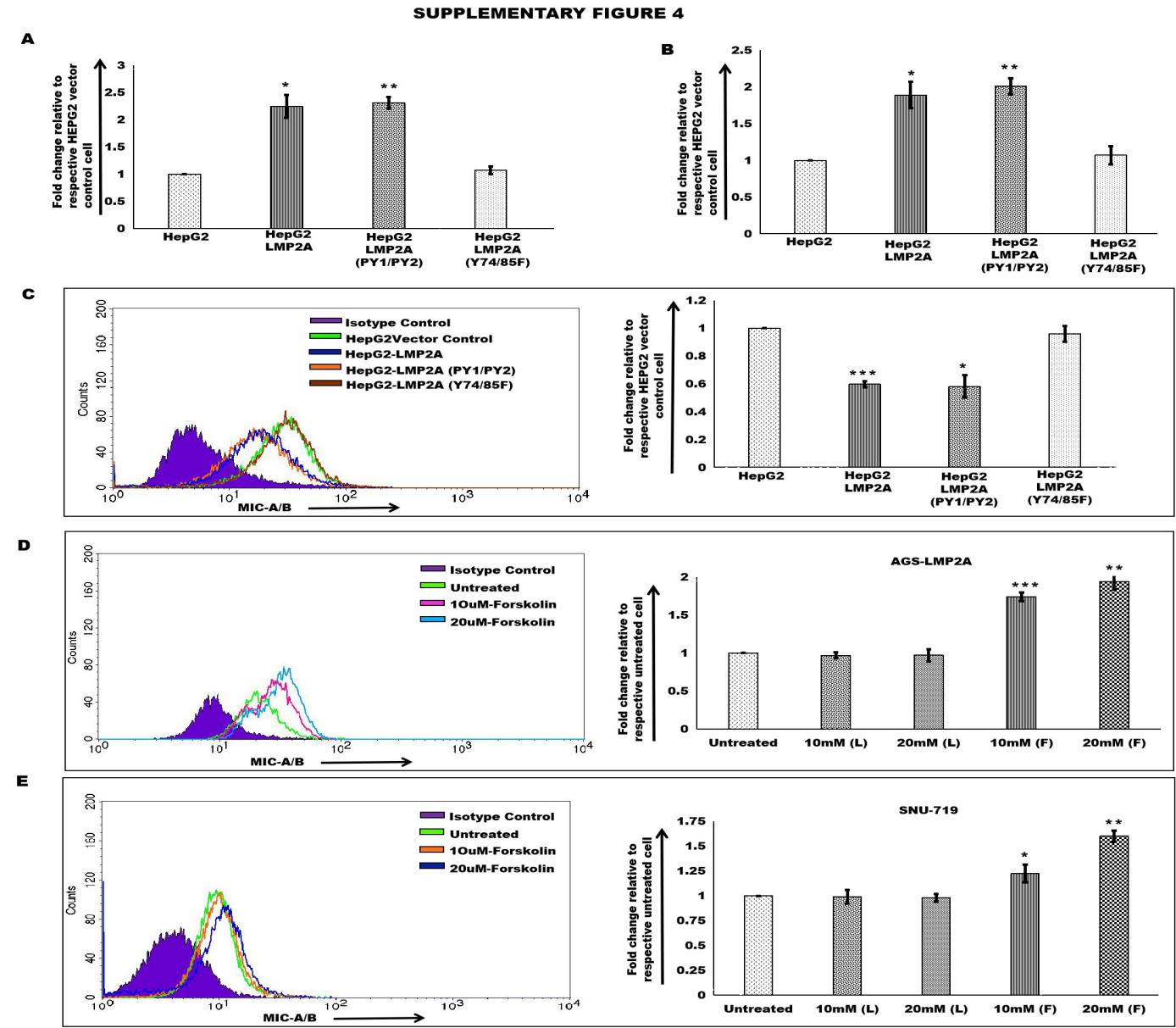


Supplementary Figure 4. (A) qRT-PCR to analyze MIC-A transcript level expression in HepG2-LMP2A, HepG2-LMP2A(PY1/PY2) and HepG2-LMP2A(Y74/85F) cells relative to HepG2 vector control cells. (B) qRT-PCR to analyze MIC-B transcript level expression in HepG2-LMP2A, HepG2-LMP2A(PY1/PY2) and HepG2-LMP2A(Y74/85F) cells relative to HepG2 vector control cells. (C) MIC-A/B surface expression upon introduction of LMP2A, mutant LMP2A(PY1/PY2) and LMP2A(Y74/85F) into HepG2 cells relative to HepG2 vector control cells. (D) MIC-A/B surface expression determination upon Forskolin ‘F’ and LY294002 ‘L’ treatment in a dose dependent manner in AGS-LMP2A cells compared to untreated cells. (E) MIC-A/B surface expression determination upon Forskolin ‘F’ and LY294002 ‘L’ treatment in a dose dependent manner in SNU-719 cells compared to untreated cells. Quantitation of results was performed by measuring median values using Cell Quest Pro software. Results are represented as mean ±s.e.m. of triplicate experiments. Data represents an average of n=3 independent experiments.*P < 0.05, **P ≤ 0.01, ***P ≤ 0.001.

Supplementary Table (1) Primer sequences used during qRT-PCR.

| ***Gene*** | ***Primer Sequence*** |
| --- | --- |
| DNMT1 (Fw) | 5’-ATGCTTACAACCGGGAAGTG-3’ |
| DNMT1 (Rv) | 5’-TGAACGCTTAGCCTCTCCAT-3’ |
| DNMT 3A (Fw) | 5’-ATCTCCAAGTCCCCATCCAT-3’ |
| DNMT 3A (Rv) | 5’-CAGCCATTTTCCACTGCTCT-3’ |
| DNMT 3B (Fw) | 5’-AGATCAAGCTCGCGACTCTC- 3’ |
| DNMT 3B (Rv) | 5’-GGCTTTCTGAACGAGTCCTG-3’ |
| HLA-A (Fw) | 5’-CCTGGGCAGTCGCACTGC-3’ |
| HLA-A (Rv) | 5’-GATTCTCCCCAGACGCCGAG-3’ |
| HLA-B (Fw) | 5’-GGACAGCCAGACCAGCAACA-3’ |
| HLA-B (Rv) | 5’-GATTCTCCCCAGACGCCGAG-3’ |
| HLA-C (Fw) | 5’-TCAGAGCCCTGGGCACTGTT-3’ |
| HLA-C (Rv) | 5’-GATTCTCCCCAGACGCCGAG-3’ |
| HLA-E (Fw) | 5’-CCACCATGGTAGATGGAACCCTC-3’ |
| HLA-E (Rv) | 5’-GCGCTTTACAAGCTGTGAGACTC-3’ |
| HLA-F (Fw) | 5’-ATCGTTGCTGGCCTTGTTGTCCTT-3’ |
| HLA-F (Rv) | 5’-GGCACAAGTGGAATTCTGCTAC-3’ |
| HLA-G (Fw) | 5’-GGAAGAGGAGACACGGAACA-3’ |
| HLA-G (Rv) | 5’-TGAGACAGAGACGGAGACAT-3’ |
| HPRT (Fw) | 5’-GACACTGGCAAAACAATGCAGAC-3’ |
| HPRT (Rv) | 5’-TGGCTTATATCCAACACTTCGTGG-3’ |
| MIC-A (Fw) | 5’-CCTTGGCCATGAACGTCAGG-3’ |
| MIC-A (Rv) | 5’-CCTCTGAGGCCTCGCTGCG-3’ |
| MIC-B (Fw) | 5’-ACCTTGGCTATGAACGTCACA-3’ |
| MIC-B (Rv) | 5’-CCCTCT GAGACCTCGCTGCA-3’ |
| UHRF1 (Fw) | 5’-TGAATGACACCATCCAGCTC-3’ |
| UHRF1 (Rv) | 5’-TCTCATCCCACATGTCCTCA-3’ |
| ULBP1 (Fw) | 5’-CAAGTGGAGAATTTAATACCCATTGAG-3’ |
| ULBP1 (Rv) | 5’-TGTTGTTTGAGTCAAAGAGGA-3’ |
| ULBP2 (Fw) | 5’-TTACTTCTCAATGGGAGACTGT-3’ |
| ULBP2 (Rv) | 5’-TGTGCCTGAGGACATGGCGA-3’ |
| ULBP3 (Fw) | 5’-CCTGATGCACAGGAAGAAGAG-3’ |
| ULBP3 (Rv) | 5’-TATGGCTTTGGGTTGAGCTAAG-3’ |
| ULBP4 (Fw) | 5’-CCTCAGGATGCTCCTTTGTGA-3’ |
| ULBP4 (Rv) | 5’-CGACTTGCAGAGTGGAAGGATC-3’ |
| ULBP5 (Fw) | 5’-TGGCCGACCCTCACTCTCT-3’ |
| ULBP5 (Rv) | 5’-CCGTGGTCCAGGTCTGAACT-3’ |
| ULBP6 (Fw) | 5’-AATCTCTTGTCCCCAGCCCT-3’ |
| ULBP6 (Rv) | 5’-GTGAGGGTCGTCTCGCCTA-3’ |

Supplementary Table (2) Sequences used for amplification of methylated products upon bisulfite modification using qRT-PCR

| ***Gene*** | ***Primer*** | ***Sequence*** | ***Size*** |
| --- | --- | --- | --- |
| HLA-AM | Forward | 5’-TAATTTCGATATTTTGGGAGGTC-3’ | 173 bp |
|  | Reverse | 5’-CCAAATAACTAAAACTACAAACGC-3’ |  |
| HLA-BM | Forward | 5’-TTTTTTTTAATTTGTGTCGG-3’ | 165bp |
|  | Reverse | 5’-ACTTTAAAACTAAAACCGCGAC-3’ |  |
| HLA-CM | Forward | 5’-TTTTTTGAATATTTATGACGCGT-3’ | 163bp |
|  | Reverse | 5’-CCTCTAAAAAAAATATAAATCCGA-3’ |  |
| GAPDH  (Methylated) | Forward | 5’-TTTTTTTATGTTGGGGTTTATATG-3’ | 142bp |
|  | Reverse | 5’-AAAACCTAAACTACATACACCCAT-3’ |  |

Supplementary Table (3) List of antibodies used in this study.

| **ANTIBODY** | **CATALOGUE NUMBER** | **MANUFACTURER** |
| --- | --- | --- |
| HLA-ABC (FACS) | 555555 | BD Biosciences |
| MIC-A/B (FACS) | 558352 | BD Biosciences |
| EBV-LMP2A (Western Blot) | ab59026 | Abcam |
| DNMT1 (Western Blot) | ab13537 | Abcam |
| DNMT3B (Western Blot) | ab13604 | Abcam |
| UHRF1 (Western Blot) | D6G8E | CST |
| IRE1alpha (Western Blot) | 3294S | CST |
| BIP (Western Blot) | 3177S | CST |
| PDI (Western Blot) | 3501S | CST |
| CHOP (Western Blot) | 2895S | CST |
| β-ACTIN (Western Blot) | ab49900 | Abcam |
| MIC-A (Western Blot) | ab150355 | Abcam |
| MIC-B (Western Blot) | ab196549 | Abcam |
